# Supplementary material for: Content-rich biological network constructed by mining PubMed abstracts
Source: BMC Bioinformatics. 2004 Oct 8;5:147. doi: 10.1186/1471-2105-5-147 (PMC528731; doi:10.1186/1471-2105-5-147)
Supplement: Additional File 2 — The original results of the above study (non-essential files are deleted to keep the file size under the limit set by BMC bioinformatics). [file 1471-2105-5-147-S2.bz2 › chilibotAdditionalFile2/dip10/684ID9215632E17999/html/CRP_CYTR.html]

 


 **CRP** and **CYTR** 
  
Found 51 abstracts in PubMed, retrieved 10.  
 

 What does Google say? 
 PDF only 
| .edu only 

---

**Interactive relationship** (e.g. stimulation, inhibition, etc)

**Neutral relationship**- Role of protein protein bridging interactions on cooperative assembly of DNA bound  **CRP**   **CytR**   **CRP**  complex and regulation of the Escherichia coli  **CytR**  regulon.  Ref: 12667072 Biochemistry, 2003
- DNA induced changes were observed also in the region involved in  **CRP**   **CytR**  interaction and in  **CRP**  intersubunit contact regions.  Ref: 10388556 J Mol Biol, 1999
- **CytR**  controls the balance between  **CRP**  mediated recruitment and activation of RNA polymerase and transcriptional repression.  Ref: 12667072 Biochemistry, 2003
- Identification of the subunit of cAMP receptor protein  [ **CRP** ]   **CRP**  that functionally interacts with  **CytR**  in  **CRP**   **CytR**  mediated transcriptional repression.  Ref: 10766824 J Biol Chem, 2000
- However, cooperativity also means that  **CytR**  can recruit  **CRP**  and, by doing so, can act as a co activator.  Ref: 10347150 J Biol Chem, 1999
- The central role of cooperativity in regulation is highlighted by the fact that binding of the inducer, cytidine, to  **CytR**  is coupled to  **CytR**   **CRP**  cooperativity.  Ref: 10347150 J Biol Chem, 1999
- Here too, we find that  **CytR**  both cooperates and competes for  **CRP**  binding.  Ref: 10347150 J Biol Chem, 1999
- Transcription is repressed by a three protein complex cAMP receptor protein  [ **CRP** ]   **CRP**   **CytR**   **CRP**  that is stabilized by cooperative interactions between  **CRP**  and  **CytR** .  Ref: 10347150 J Biol Chem, 1999
- At promoters of the Escherichia coli  **CytR**  regulon, the cAMP receptor protein  [ **CRP** ]   **CRP**  interacts with the repressor  **CytR**  to form transcriptionally inactive  **CRP**   **CytR**  promoter or  **CRP**  2  **CytR**  promoter complexes.  Ref: 10766824 J Biol Chem, 2000
- Here, using oriented heterodimer analysis, we show that only one subunit of the  **CRP**  dimer, the subunit proximal to  **CytR** , functionally interacts with  **CytR**  in  **CRP**   **CytR**  promoter and  **CRP**  2  **CytR**  promoter complexes.  Ref: 10766824 J Biol Chem, 2000
- We have suggested that this variability affects the flexibility of  **CytR**  DNA contacts, thereby affecting the critical protein protein interactions between  **CytR**  and the cAMP receptor protein  [ **CRP** ]   **CRP**  that underlie differential repression and activation of  **CytR**  regulated genes.  Ref: 11866516 J Mol Biol, 2002
- In the deoP2 promoter of Escherichia coli, a transcription activator, cAMP  **CRP** , binds at two sites, centered at 41.5 and 93.5 from the start site of transcription, while a repressor,  **CytR** , binds to a space between the two cAMP  **CRP**  complexes.  Ref: 11574471 EMBO J, 2001
- The  **CytR**  anti activator diverges from the other family members by relying on the cooperative DNA binding with the global regulator  **CRP** .  Ref: 10329134 J Mol Biol, 1999
- Cooperative interactions between  **CytR** , when bound to an operator CytO located upstream of a  **CytR**  regulated promoter, and  **CRP** , when bound to flanking tandem promoters, are critical to the regulatory role of  **CytR** .  Ref: 12667072 Biochemistry, 2003
- Recently, we showed that  **CytR**  binds specifically to multiple sites in the E. coli deoP promoter, thereby providing competition for  **CRP**  binding to  **CRP**  operator site 1 CRP1 and CRP2 as well as cooperativity.  Ref: 10347150 J Biol Chem, 1999
- The unlinked operons that comprise the Escherichia coli  **CytR**  regulon are controlled coordinately through interactions between two gene regulatory proteins, the cAMP receptor protein  [ **CRP** ]   **CRP**  and the cytidine repressor  **CytR** .  Ref: 12667072 Biochemistry, 2003

**Non-interactive relationship** (e.g. studied together, co-existance, homology, etc.)

- The data presented suggest that the expression of the P2 promoter, unlike that of P1, is not subjected to regulation with participation of the  **CytR**  protein and the cAMP  **CRP**  complex.  Ref: 12068544 Genetika, 2002
- Cooperativity with  **CRP**  at CRP1 is nearly eliminated, but the effect on  **CytR**  CRP2 cooperativity is negligible.  Ref: 10347150 J Biol Chem, 1999
- Our results provide information about the architecture of  **CRP**   **CytR**  promoter and  **CRP**  2  **CytR**  promoter complexes and rule out the proposal that masking of activating region 2 of  **CRP**  is responsible for the transcriptional inactivity of the complexes.  Ref: 10766824 J Biol Chem, 2000
- Despite only weak association between  **CytR**  and  **CRP**  in solution, our results demonstrate the formation of a relatively stable complex in which the Core domain forms a protein bridge between tandem  **CRP**  dimers when bound to either udpP or deoP2.  Ref: 12667072 Biochemistry, 2003
- The mechanisms for the cAMP  **CRP**  mediated transcription activation and  **CytR**  mediated transcription repression were investigated in vitro using purified components.  Ref: 11574471 EMBO J, 2001
- The effect of the competition at this promoter is to negate the role of  **CytR**  in recruiting  **CRP** .  Ref: 10347150 J Biol Chem, 1999
- However, this cytidine mediated effect varies among promoters, suggesting that coupling between cytidine binding to  **CytR**  and  **CytR**   **CRP**  association is sensitive to promoter structure.  Ref: 12667072 Biochemistry, 2003
- To investigate the chemical and structural basis for these effects, we investigated how cytidine binding affects association between  **CytR**  and  **CRP**  in solution and how it affects the binding of  **CytR**  deletion mutants lacking the DNA binding HTH domain, with tandem  **CRP**  dimers bound to either udpP or deoP2.  Ref: 12667072 Biochemistry, 2003
- After introduction of mutations affecting binding sites for  **CRP** , the negative effect of the  **CytR**  protein on promoter transcription was fully abolished.  Ref: 12391883 Genetika, 2002
- The mechanism of repression by  **CytR**  proposed in this study is that the cAMP  **CRP**  bound at 41.5 undergoes an allosteric change upon direct interaction with  **CytR**  such that it no longer maintains a productive interaction with the N terminal domain of alpha, but instead acts as a repressor to interfere with RNA polymerase acting on deoP2.  Ref: 11574471 EMBO J, 2001
- In mutants with an improved 10 region, a partial relief from the control of the cAMP  **CRP**  transcription activation complex occurred, and the negative  **CytR**  repressor regulation was reduced.  Ref: 12391883 Genetika, 2002
- Here, we have used quantitative footprint and mobility shift analysis to investigate  **CRP**  and  **CytR**  binding to the E. coli udp promoter.  Ref: 10347150 J Biol Chem, 1999
- Sequences similar to the sigma 70 promoters P1, P4 and P5, to the sigma E promoter P3 and to boxes DnaA1, DnaA2, cAMP receptor protein  [ **CRP** ]   **CRP**  boxes CRP1, CRP2 and box  **CytR**  present in Escherichia coli K12, were identified in sequences of closely related bacteria such as E.coli, Shigella flexneri, Salmonella enterica serovar Typhimurium, Citrobacter freundii, Enterobacter cloacae and Klebsiella pneumoniae.  Ref: 11139607 Nucleic Acids Res, 2001
- These findings demonstrate that direct coupling between cytidine binding to  **CytR**  and  **CytR**   **CRP**  association does not account for promoter specific effects on cooperativity.  Ref: 12667072 Biochemistry, 2003
- In Process Citation Site directed mutagenesis was conducted in the regulatory region of the Escherichia coli udp gene at promoter sites responsible for binding regulatory proteins  **CRP**  and  **CytR**  as well as RNA polymerase.  Ref: 12391883 Genetika, 2002
